# Incidence of lymph node metastases in pT1 chemo- and radiotherapy-naïve esophageal and gastric cancer in the Nordic countries: a retrospective multinational cohort analysis

**DOI:** 10.1093/dote/doag105

**Published:** 2026-09-24

**Authors:** David Edholm, Joonas H Kauppila, Michael P Achiam, Oliver Nørholm Kempf, Tobias Hauge, Mats Lindblad, Tom Mala

**Affiliations:** Department of Surgery, Linköping University, Linköping, Sweden; Department of Surgery, Oulu University Hospital and University of Oulu, Oulu, Finland; Department of Transplantation and Digestive Diseases, Copenhagen University Hospital Rigshospitalet, Copenhagen, Denmark; Department of Clinical Medicine, University of Copenhagen, Copenhagen, Denmark; Department of Transplantation and Digestive Diseases, Copenhagen University Hospital Rigshospitalet, Copenhagen, Denmark; Department of Gastrointestinal Surgery, Oslo University Hospital and Institute of Clinical Medicine, University of Oslo, Oslo, Norway; Center for Digestive Diseases, Karolinska University Hospital and the Division of Surgery and Oncology, Department of Clinical Science, Intervention and Technology (CLINTEC), Karolinska Institutet, Stockholm, Sweden; Department of Gastrointestinal Surgery, Oslo University Hospital and Institute of Clinical Medicine, University of Oslo, Oslo, Norway

**Keywords:** early cancer, esophageal cancer, gastric cancer, lymphatic metastasis

## Abstract

Although endoscopic resection is reported to be safe for early-stage esophagogastric cancer, uncertainty remains regarding oncological efficacy, particularly for submucosal lesions. The aim of this study was to assess the frequency of lymph node metastasis (LNM) and survival in patients operated for chemo- and radiotherapy-naïve pT1 esophagogastric cancer. Patients who underwent surgical resection for esophageal or gastric pT1 adenocarcinoma or squamous cell carcinoma without neoadjuvant oncologic treatment were included. Data were retrospectively extracted from registries in Denmark (2013–2021), Norway (2007–2024), Sweden (2012–2022), and Finland (2000–2016). The primary outcome was the frequency of LNM in resected specimens from patients operated for pT1 esophagogastric cancer. Secondary outcomes included: 5-year overall survival, number of lymph nodes harvested, and risk factors associated with LNM and mortality. 1647 patients with pT1 tumors were identified, of whom 949 (58%) did not receive neoadjuvant treatment; 413 (43%) had esophagectomy and 536 (56%) gastrectomy. 145 (15%) patients had LNM identified in the resected specimens for esophagectomies, the LNM rate was 3% for pT1a and 20% for pT1b, and for gastrectomies, 7% for pT1a and 21% for pT1b. The median follow-up was 62 months (interquartile range: 33–102) and 5-year overall survival was 81% for patients with pT1a tumors and 65% for those with pT1b tumors. Among patients with pT1b, the overall survival was 69% without LNM and 51% with LNM. Depth of tumor invasion was associated with risk of LNM. A high proportion of patients had LNM after resection for chemo- and radiotherapy-naïve pT1 esophagogastric cancer.

## BACKGROUND

Surgical resection remains the cornerstone of curative treatment for esophagogastric cancer. However, this treatment is associated with significant morbidity and mortality, making an organ-sparing endoscopic resection an appealing alternative for patients with very early cancers. A low risk of lymph node metastases (LNM) is essential for endoscopic resection to be considered an appropriate oncological treatment option for patients otherwise eligible for surgery.

The risk of LNM following endoscopic resections of high-risk early gastric cancer has been reported to be 8% in a South Korean study.^1^ Although not directly comparable, surgical series from Europe and the United States report significantly higher risks of LNM (13–20%) in early esophagogastric cancer, raising concerns about whether the findings from Asian studies can be applied to Western patients.^2–5^ Survival rates for patients with early gastric cancer are lower in Western countries than in Japan, suggesting that the disease, patients, treatment, and pathological evaluation may differ in various aspects across regions.^3^^,^^6^

A major challenge in preoperative, clinical staging of early esophagogastric cancer is the low sensitivity and specificity of imaging such as computed tomography (CT), endoscopic ultrasound (EUS), and positron emission tomography (PET) for identifying LNM.^7^ Recent international guidelines specify criteria for considering an endoscopic resection as potentially curative.^8–10^ As a result, these guidelines have become standard reference in most centers’ multidisciplinary tumor board discussions when giving recommendation on the need for additional treatment following endoscopic resection. Factors associated with an increased risk of LNM in esophageal cancer include undifferentiated tumors,^11^ submucosal invasion,^12^ tumor size greater than 15 mm,^13^ lymphovascular^12^ and perineural invasion.^14^ For gastric cancer, similar risk factors have been identified.^15^

The aim of this study was to investigate the frequency of LNM and overall survival in a large Nordic cohort of patients with early, chemo- and radiotherapy-naïve esophagogastric cancer treated with esophagectomy or gastrectomy, based on findings from histopathological specimen evaluations.

## METHODS

### Study design

This is a retrospective analysis utilizing registry data from Finland, Denmark, Sweden, and Norway on patients who underwent surgery for chemo- and radiotherapy-naïve pathologically T1 (pT1) esophagogastric cancer.

### Patients

Patient data were analyzed and merged from the national registries of Finland (2000–2016),^16^ Denmark (2013–2021), and Sweden (2012–2022). A regional registry for southern Norway, including more than half of the national population, was utilized for data collection (2007–2024). For gastric cancer, this registry included the majority of patients who underwent surgery in the region during the later years of the study period; however, coverage was less comprehensive in the earlier years. All patients who underwent esophagectomy for esophageal adenocarcinoma or squamous cell carcinoma (SCC), or gastrectomy for gastric adenocarcinoma in whom the histopathological assessment resulted in a tumor stage of pT1 according to the AJCC Tumor-Node-Metastasis (TNM) Classification System were included. According to the AJCC 8^th^ edition^17^ pT1a refers to cancer that invades the lamina propria or muscularis mucosae, whereas pT1b describes cancer that invades the submucosa.^18^ To minimize variability in tumor classification across centers, patients were stratified based on the type of surgical resection performed—either esophagectomy or gastrectomy. Patients who had received neoadjuvant oncologic treatment were excluded.

### Outcome measures

The primary outcome was the frequency of LNM in patients operated for pT1 esophagogastric cancer. Secondary outcomes included 5-year overall survival, number of lymph nodes harvested, risk factors associated with LNM, and mortality.

### Statistical analysis

Patients were followed up from the date of surgery until death, emigration, or the end of follow-up, whichever occurred first. Crude survival curves were displayed using Kaplan–Meier plots. Cox proportional hazard models were used to estimate hazard ratios for death (HR) with 95% confidence intervals (CI) in both univariable and multivariable models. Associations between exposures, mortality, and LNM were analyzed using univariable and multivariable logistic regression, yielding odds ratios (ORs) with 95% CIs. Variables with *P*-values <0.1 in univariable analysis were included in the multivariable model; the *P* < 0.1 threshold was chosen to reduce the risk of statistical type II error. The following covariates were included: age (continuous variable), sex (male or female), fitness according to the American Society of Anesthesiologists^19^ (ASA 1, 2, 3, or 4), pathological T-stage (pT1a or pT1b), type of surgery (esophagectomy or gastrectomy), tumor histology (adenocarcinoma or SCC). For the survival analysis, pathological N-stage (pN0, pN1, pN2, or pN3) was added. Missing data were handled by patient exclusion from analysis. Survival status was fully captured, using a unique personal identification number assigned to individuals in each country.

Statistical analysis was done using STATA/IC 15.1 SE (StataCorp, College Station, Texas, USA).

### Ethics

Patient data were handled according to local legislation at each participating center and anonymous data were merged into one dataset. The study complies with the Declaration of Helsinki.

## RESULTS

### Patient characteristics

1647 patients with pT1 tumors were identified, of whom 949 (58%) did not receive neoadjuvant treatment and were included in this study. Esophagectomy was performed in 413 (43%) and gastrectomy in 536 (56%). In patients who underwent esophagectomy, 324 (78%) had adenocarcinoma, 75 (18%) SCC, and 14 patients (3%) had an undefined carcinoma. In comparison, 528 patients (99%) who underwent gastrectomy had adenocarcinoma, while eight patients (1%) had undefined carcinoma. Overall, 341 (36%) patients had pT1a tumors and 608 (64%) pT1b tumors. Patient characteristics are given in Table 1.

**Table 1:** Demographic data on 949 patients with chemo- and radiotherapy-naïve pT1 gastroesophageal cancer

	All *n* = 949		Esophagectomy *n* = 413		Gastrectomy *n* = 536	
Age, years (median, IQR)	70 (61–77)		68 (60–74)		72 (62–79)	
	*n*	%	*n*	%	*n*	%
Sex						
Male	629	66%	327	79%	302	56%
Female	320	34%	86	21%	234	44%
American Society of Anesthesiologist class						
1	117	12%	53	13%	64	12%
2	360	38%	179	43%	181	34%
3	348	37%	139	34%	209	39%
4	58	6%	15	4%	43	8%
Missing	66	7%	27	7%	39	7%
Type of surgery						
Esophagectomy	413	43%	413	100%		
Gastrectomy	536	56%			536	100%
Histology						
Adenocarcinoma	852	90%	324	78%	528	99%
Squamous cell carcinoma	75	8%	75	18%	0	0%
Undifferentiated carcinoma	22	2%	14	3%	8	1%
pT						
pT1a	341	36%	136	33%	205	38%
pT1b	608	64%	277	67%	331	62%
pN						
pN0	804	85%	353	86%	451	84%
pN1	95	10%	44	11%	51	9%
pN2	40	4%	11	3%	29	5%
pN3	10	1%	5	1%	5	1%
Involved nodes	145	15%	60	15%	85	16%
Lymph node yield (median) (IQR)	17 (9–26)		18 (10–28)		17 (8–24)	

IQR, Interquartile range; pT, pathological tumor stage; pN, pathological nodal stage.

### Lymph node evaluations

Of the 949 included patients, 145 (15%) had LNM: 19 (6%) with pT1a tumors and 126 (21%) with pT1b tumors (Table 2). The median number of lymph nodes examined was 17 (interquartile range (IQR): 9–26) and the proportion of patients with a lymph node yield of 15 or more was 61%. Less than 15 lymph nodes were examined in 33% of patients with pT1a tumors and 43% of patients with pT1b*.*

**Table 2:** Lymph nodes status according to tumor location, histology, and pathological stage from 949 patients after esophagogastric resection

Pathological stage	No involved nodes (*n*)	Involved nodes (*n*)
All patients		
1a	94% (322)	6% (19)
1b	79% (482)	21% (126)
All esophagectomies		
1a	97% (132)	3% (4)
1b	80% (221)	20% (56)
Esophagectomies—adenocarcinoma only		
1a	97% (106)	3% (3)
1b	81% (175)	19% (40)
Esophagectomies—squamous cell carcinoma only		
1a	95% (21)	5% (1)
1b	75% (40)	25% (13)
All gastrectomies		
1a	93% (190)	7% (15)
1b	79% (261)	21% (70)

### Assessment of lymph node metastasis in esophagectomy specimens

In all patients who underwent esophagectomy, pT1a was associated with 3% risk of LNM and pT1b with a 20% risk of LNM, the median lymph node yield was 18 (IQR: 10–28). Less than 15 lymph nodes were examined in 37% of all esophagectomies. For patients with adenocarcinoma, pT1a was associated with 3% risk of LNM and pT1b with a 19% risk of LNM. For SCC, pT1a was associated with 5% risk of LNM and pT1b with a 25% risk of LNM (Table 2). Multivariable analysis showed that only pathological T1b remained an independent predictor of increased risk of LNM (OR = 10.62, 95% CI [3.25–34.76]), female gender was an independent predictor of decreased risk of LNM (OR = 0.30, 95% CI [0.11–0.79]) (Table 3).

**Table 3:** Risk of nodal involvement, expressed as odds ratio (OR) with 95% confidence intervals (CI) in 413 patients with chemo- and radiotherapy-naïve pT1 tumors undergoing esophagectomy

	Univariable		Multivariable	
	OR	(95% CI)	OR	(95% CI)
Sex				
Male	1.00	(reference)	**1.00**	**(reference)**
Female	0.31	(0.12–0.78)	**0.30**	**(0.11–0.79)**
Age at diagnosis				
(continuous)	1.04	(1.01–1.07)	1.03	(0.99–1.06)
American Society of Anesthesiologist class				
1	1.00	(reference)		
2	0.83	(0.34–1.98)		
3	1.23	(0.51–2.93)		
4	0.40	(0.05–3.49)		
Histology				
Adenocarcinoma	1.00	(reference)		
Squamous cell carcinoma	1.50	(0.77–2.91)		
Pathological T-stage				
pT1a	1.00	(reference)	**1.00**	**(ref.)**
pT1b	8.36	(2.96–23.59)	**10.62**	**(3.25–34.76)**

Variables with significant result in the multivariable analysis are shown in bold.

### Assessment of lymph node metastasis in gastrectomy specimens

In patients with gastric cancer, pT1a was associated with 7% risk of LNM and pT1b with a 21% risk of LNM; the median lymph node yield was 17 (IQR: 8–24) (Table 2). Less than 15 lymph nodes were examined in 41% of gastrectomies. In the multivariable analysis, female gender (OR = 1.70, 95% CI [1.06–2.73]) and pathological T1b (OR = 3.45, 95% CI [1.91–6.23]) were independent predictors of increased risk of LNM (Table 4).

**Table 4:** Risk of nodal involvement, expressed as odds ratio (OR) with 95% confidence intervals (CI) in 536 patients with chemo- and radiotherapy-naïve pT1 tumors undergoing gastrectomy

	Univariable		Multivariable	
	OR	(95% CI)	OR	(95% CI)
Sex				
Male	1.00	(reference)	1.00	(reference)
Female	1.65	(1.04–2.63)	**1.70**	**(1.06–2.73)**
Age at diagnosis				
(continuous)	1.01	(0.99–1.02)		
American Society of Anesthesiologist class				
1	1.00	(reference)		
2	1.31	(0.59–2.92)		
3	1.10	(0.49–2.45)		
4	0.99	(0.33–3.01)		
Pathological T-stage				
pT1a	1.00	(reference)	1.00	(ref.)
pT1b	3.39	(1.88–6.11)	**3.45**	**(1.91–6.23)**

Variables with significant result in the multivariable analysis are shown in bold.

### Overall survival and risk factors associated with mortality

After a median follow-up of 62 months (IQR: 33–102), the overall 5-year survival for the entire cohort was 71%. For patients with pT1a tumors 5-year survival was 81% and for pT1b 65%.

In patients undergoing esophagectomy, the 5-year overall survival was 82% in those with pT1a tumors, compared to 63% in patients with pT1b tumors.

In patients undergoing gastrectomy, the 5-year overall survival was 72% for all pT1 tumors, and 79% in patients with pT1a tumors and 67% in patients with pT1b tumors.

**Fig. 1:**
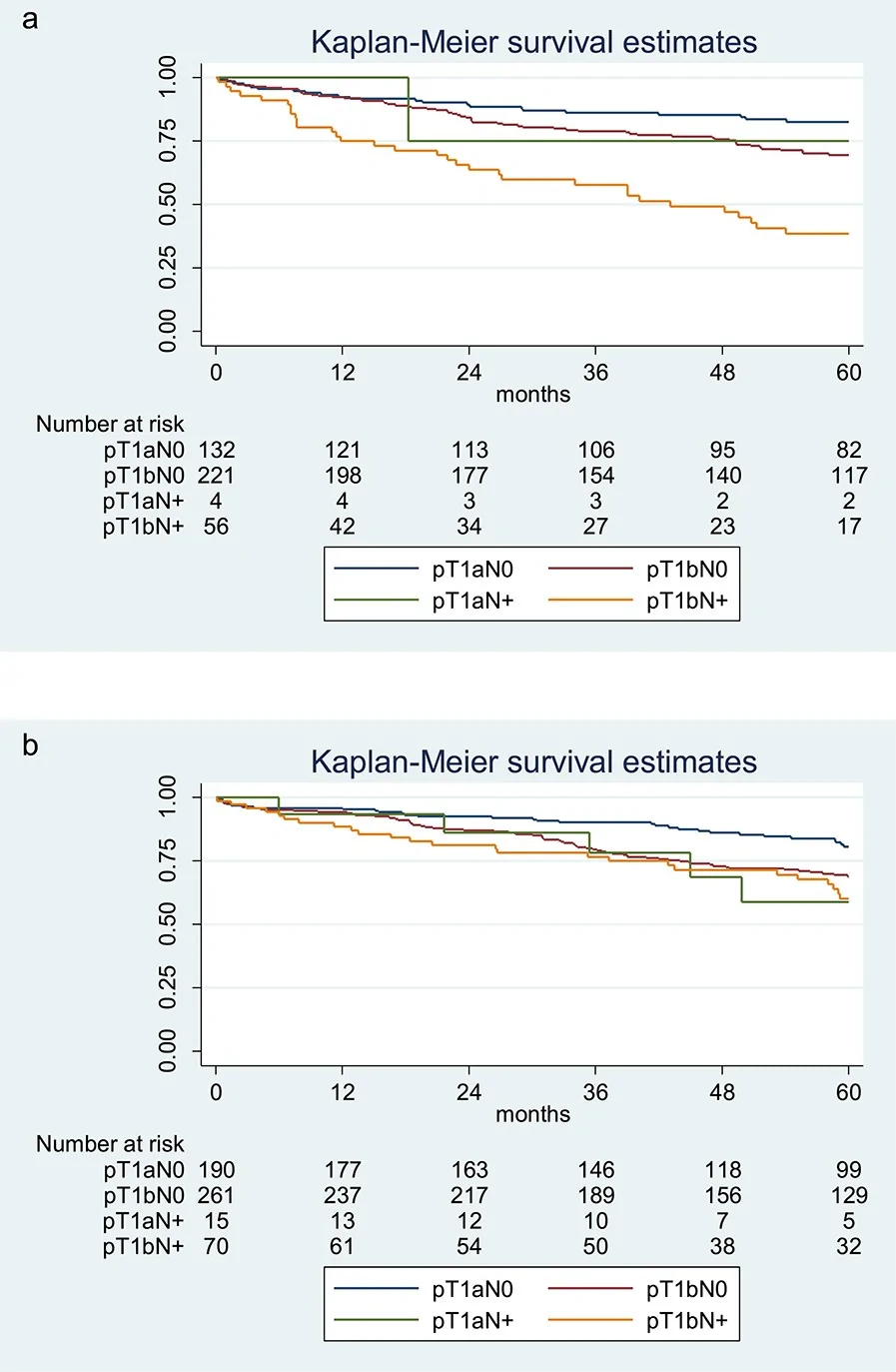
Five-year overall survival of treatment-naïve pT1 esophagogastric cancer patients stratified by T-stage and lymph node status. Kaplan–Meier survival curves for (a) patients undergoing esophagectomy (*n* = 413) and (b) patients undergoing gastrectomy (*n* = 536). Curves compare individuals with pT1a disease (5-year survival 82% for esophagus, 79% for stomach) against pT1b disease (5-year survival 63% for esophagus, 67% for stomach) further sub-stratified by pN status.

Figure 1a and 1b illustrate overall survival according to pT-stage and lymph nodes status for esophagectomy and gastrectomy, respectively.

In the multivariable model for patients undergoing esophagectomy (Table 5), increasing age, SCC, pT1b, and pN+ stage were associated with increased risk of death within five years after resection, while female sex was associated with decreased risk.

**Table 5:** Risk of death, expressed as hazard ratio (HR) with 95% confidence intervals (CI) in 413 patients with chemo- and radiotherapy-naïve pT1 tumors who underwent esophageal cancer resection

	Univariable		Multivariable	
	HR	(95% CI)	HR	(95% CI)
Sex				
Male	1.00	(reference)	1.00	(reference)
Female	0.56	(0.33–0.95)	**0.47**	**(0.27–0.84)**
Age at diagnosis				
(continuous)	1.03	(1.02–1.06)	**1.03**	**(1.01–1.05)**
American Society of Anesthesiologist class				
1	1.00	(reference)		
2	0.72	(0.40–1.29)		
3	1.19	(0.68–2.11)		
4	1.05	(0.38–2.86)		
Histology				
Adenocarcinoma	1.00	(reference)	1.00	(reference)
Squamous cell carcinoma	1.49	(0.96–2.29)	**1.85**	**(1.17–2.92)**
Pathological T-stage				
pT1a	1.00	(reference)	1.00	(reference)
pT1b	2.25	(1.42–3.54)	**1.94**	**(1.18–3.17)**
Pathological N-stage				
pN0	1.00	(reference)	1.00	(reference)
pN1	2.78	(1.76–4.38)	**1.99**	**(1.22–3.24)**
pN2	3.22	(1.30–7.97)	**1.89**	**(0.68–5.22)**
pN3	4.38	(1.60–11.97)	**3.61**	**(1.30–9.98)**

Variables with significant result in the multivariable analysis are shown in bold

In the multivariable model for patients undergoing gastrectomy (Table 6), increasing age, ASA class >2, pT1b and pN3 stage were associated with increased risk of death within five years after resection.

**Table 6:** Risk of death, expressed as hazard ratio (HR) with 95% confidence intervals (CI) in 536 patients with chemo- and radiotherapy-naïve pT1 tumors who underwent gastrectomy for cancer

	Univariable		Multivariable	
	HR	(95% CI)	HR	(95% CI)
Sex				
Male	1.00	(reference)		
Female	0.75	(0.53–1.07)		
Age at diagnosis				
(continuous)	1.06	(1.04–1.08)	**1.04**	**(1.03–1.07)**
American Society of Anesthesiologist class				
1	1.00	(reference)	1.00	(reference)
2	1.17	(0.53–2.60)	0.84	(0.37–1.90)
3	2.82	(1.35–5.89)	**1.62**	**(0.76–3.43)**
4	6.41	(2.89–14.22)	**3.05**	**(1.32–7.05)**
Pathological T-stage				
pT1a	1.00	(reference)	1.00	(reference)
pT1b	1.75	(1.19–2.56)	**1.30**	**(0.85–2.01)**
Pathological N-stage				
pN0	1.00	(reference)	1.00	(reference)
pN1	1.46	(0.87–2.44)	1.55	(0.88–2.71)
pN2	1.84	(0.99–3.42)	1.42	(0.73–2.77)
pN3	2.70	(0.66–10.96)	**5.50**	**(1.28–23.57)**

Variables with significant result in the multivariable analysis are shown in bold.

## DISCUSSION

The main finding of this retrospective Nordic cohort study was that 15% of patients operated for chemo- and radiotherapy-naïve pT1 esophagogastric cancer had LNM. Notably, overall LNM occurred in 6% of patients operated for pT1a tumors and 21% of those with pT1b tumors, with lower overall 5-year survival observed in the latter group.

The incidences of LNM were higher than in a Korean study of 147 patients who underwent gastrectomy following endoscopic resection of high-risk early gastric cancer (8%).^1^ Our findings might not be directly comparable as the Korean study only included preoperative cN0 patients. In a Chinese study of 223 patients operated for esophageal pT1 SCC, a LNM rate of 16% was found, which is comparable to our findings, despite the predominance of adenocarcinoma in our study.^20^

When subclassified according to type of surgical resection and histology, LNM occurred in 3% of our patients operated with esophagectomy for a pT1a adenocarcinoma and in 19% of those with pT1b adenocarcinoma. These findings are in line with recent observations from a meta-analysis of 20 studies from Western countries involving 2264 patients resected for pT1 esophageal adenocarcinoma.^21^ The meta-analysis demonstrated a 4.2% risk of LNM for stage pT1a and a 23.2% risk for pT1b esophageal adenocarcinoma, respectively.^21^

The overall 5-year survival rate in our series was 81% for patients with pT1a cancer and 65% in patients with pT1b tumors, highlighting the important association between tumor depth invasion and survival. These findings are consistent with those of Pennathur *et al*., who reported a 5-year overall survival rate of 73% for T1a and 60% pT1b tumors, respectively.^22^

Notably, Choi *et al*. found that survival was associated with ethnicity, with a 5-year survival of 88% for T1a gastric cancer among Asians compared to 77% for Caucasians (*P* < 0.01).^23^

A recent meta-analysis reported that CT has a pooled sensitivity of 0.57 and a specificity of 0.92 in detecting LNM in patients with gastric cancer.^7^ Similarly, PET–CT underestimates the number of LNM in patients with esophageal cancer, with a pooled sensitivity of 0.65 and specificity of 0.81.^24^ Due to low sensitivity and specificity in preoperative evaluation of LNM our findings suggest meticulous histopathological evaluation of the endoscopically removed specimen, with particular attention to submucosal depth of tumor infiltration, before accepting endoscopic resection as the final treatment recommendation in patients with pT1b cancer.^21^ Submucosal tumor infiltration depth, lymphovascular infiltration, and grade of tumor differentiation have been associated with increasing risk of LNM.^10^^,^^25^ The study findings underline the need for improved preoperative staging both in regard to resection strategy and perioperative oncologic treatment.

A recent retrospective study on endoscopic resection in 106 patients with high risk T1a and T1b esophageal adenocarcinoma, found endoscopic resection to be a valid alternative to surgery with a low metastatic risk.^26^ The study included patients from 11 tertiary referral centers in Europe and Australia and found an annual risk of metastatic events of 0.9% for low risk T1b tumors and 3.9% for high-risk T1b tumors with a median time to metastasis of 29 months. Following endoscopic resection, 26 of the patients underwent esophagectomy after a median of 2 months. The remaining 80 patients underwent endoscopic surveillance. After a median follow-up of 47 months, 15% (*n* = 4) in the surgical group had LNM and 4% (*n* = 1) had distant metastasis, compared to 5% (*n* = 4) and 1% (*n* = 1), respectively, in the endoscopic group. However, sample size was low, and the study was retrospective with potential selection biases. The findings are inconsistent with the findings of the meta-analysis from Nguyen *et al*., and from our observations of high rates of LNM in T1b tumors from a large, and unselected, chemo- and radiotherapy-naïve cohort following surgery.^21^ The ongoing prospective and multicenter PREFER study on LNM following endoscopic resection for high-isk T1a and T1b esophageal adenocarcinoma is expected to provide more robust data. Based on an interim analysis of 120 patients with a median follow-up of 22 months, LNM was observed in 5% and exclusively in patients with T1b tumors. All patients who developed LNM underwent curative surgery and no distant metastases were found.^27^

These discrepancies highlight the need for more robust evidence and long-term outcomes before potentially endorsing the widespread use of endoscopic resection as standalone treatment in patients with T1b esophagogastric cancers, particularly in Nordic populations. Due to the high reported rates of LNM, neoadjuvant therapy for patients with T1b tumors has been proposed.^28^

As this is a retrospective study, limitations must be acknowledged. This study merges both esophageal and gastric cancer, including adenocarcinoma and SCC, with unknown preoperative lymph node status, making the groups potentially heterogeneous and biased. Further, this study only includes a selected group of patients who were resected and whose histopathological assessment confirmed pT1 tumors. This excludes superficial tumors removed by endoscopic resection and might introduce a selection bias potentially affecting the distribution of positive and negative lymph nodes. Nevertheless, this could mimic the situation when evaluating the endoscopically resected specimen, whether to be satisfied or to proceed with esophagectomy or gastrectomy to increase the chance for cure. Specifically, the invasion depth may be underestimated in surgical specimens compared to endoscopic specimens, likely due to more extensive histopathological examination in the latter group.^29^ Histopathological evaluations of the resected specimens were not standardized in our study that may have impacted classification of T1a and T1b status. Furthermore, lymphovascular infiltration, grade of tumor differentiation, level of submucosal infiltration status were not available. As data on lymphovascular invasion, tumor grade, and detailed invasion depth were not available, risk stratification according to current European and Asian guideline criteria was not possible in the present study, and our findings should not be interpreted as a validation or refutation of those criteria.

Our study cannot be used to estimate the risk of LNM in patients who undergo endoscopic resection as preoperative lymph node status was not available. The number of lymph nodes examined was less than 15 in 40% of patients, increasing the risk of understaging; thus, the true rate of LNM in this series may be higher than reported. Variation in lymph node yield likely reflects differences in surgical technique and pathological processing across centers and time periods. During the study period, only very few patients would have been eligible for endoscopic resection as this was not standard at the time. This may thus not represent a selection bias.

Strengths of the study include the large sample size based on data from four comprehensive Nordic registries, which improves the generalizability of findings. To our knowledge, this is the most extensive population-based study evaluating the risk of LNM in patients with chemo- and radiotherapy-naïve gastroesophageal pT1 cancer. Other strengths of the study include the extensive population coverage, which minimizes selection bias, and the complete follow-up for survival analyses.

In this large retrospective Nordic cohort of surgically treated patients, 15% of those with chemo- and radiotherapy-naïve pT1 esophagogastric cancer had lymph node metastases, rising to 20–21% in pT1b tumors. Nodal involvement was associated with significantly worse 5-year overall survival. These findings characterize the burden of nodal disease in Western pT1 patients undergoing surgery, and underscore the importance of meticulous histopathological evaluation and multidisciplinary staging. Prospective studies including endoscopically treated patients are needed to inform treatment selection and the role of endoscopic resection in this population.

## Notes

Specific author contributions: David Edholm, Tom Mala; Formal analysis: David Edholm; Writing—original draft: David Edholm, Tom Mala; Writing—review & editing: Joonas H Kauppila, Michael Patrick Achiam, Oliver Nørholm Kempf.
